# Maternal inflammation disrupts transplacental antibody transfer in pregnant individuals with and without HIV

**DOI:** 10.3389/fimmu.2026.1879810

**Published:** 2026-07-28

**Authors:** Biana Bernshtein, Audrey Butler, Drucilla J. Roberts, Raymond Atwine, Julian Adong, Galit Alter, Joseph Ngonzi, Katharine F. B. Correia, Lisa M. Bebell

**Affiliations:** 1 The Shmunis School of Biomedicine and Cancer Research, The George S. Wise Faculty of Life Sciences, Tel Aviv University, Tel Aviv, Israel; 2 Ragon Institute of Massachusetts General Hospital (MGH), MIT and Harvard, Cambridge, MA, United States; 3 Department of Pathology, Massachusetts General Hospital, Boston, MA, United States; 4 Harvard Medical School, Boston, MA, United States; 5 Faculty of Medicine, Mbarara University of Science and Technology, Mbarara, Uganda; 6 Department of Mathematics and Statistics, Amherst College, Amherst, MA, United States; 7 Division of Infectious Diseases, Department of Medicine, and Center for Global Health, Massachusetts General Hospital, Boston, MA, United States

**Keywords:** Africa, fetal, HIV-exposed, maternal-child, newborn, pregnancy, Uganda, women

## Abstract

**Introduction:**

Maternally transferred immunity plays a critical role in protecting infants from pathogens during the vulnerable period before their own immune system matures. Maternal IgG antibodies cross the placenta and circulate in the infant's body for up to six months, providing protection against infection. Despite the importance of transplacental antibody transfer, the mechanisms regulating this process remain poorly understood, including the maternal and placental factors that influence transfer efficiency.

**Methods:**

We analyzed maternal and umbilical cord plasma samples from 354 mother-baby dyads in Uganda, of whom 176 were pregnant individuals with HIV taking antiretroviral therapy. We measured IgG antibody levels, antibody binding to Fc receptors, antibody glycosylation, and cytokine levels in both maternal and umbilical cord compartments, and we evaluated placental inflammatory states with placental histology. We used this comprehensive dataset to define correlations between transplacental antibody transfer efficiency, placental inflammation, and cytokine levels in maternal and umbilical cord plasma.

**Results:**

We found that antibody transfer efficiency was not impacted by histologic placental inflammation, but that higher maternal plasma levels of the pro-inflammatory cytokines TNF-α and IL-6 correlated with reduced transplacental antibody transfer efficiency.

**Discussion:**

Our findings reveal the impact of maternal systemic inflammation on antibody transfer across the placenta and have implications for maternal immunization strategies.

## Introduction

During the first months of life while the infant’s immune system matures, maternally-transferred immunity provides critical protection against infection. Antibodies transferred across the placenta circulate in the infants’ body for up to 6 months after birth to protect against infection (1, 2). Transplacentally-transferred antibodies can be naturally acquired – through spontaneous infection in the pregnant individual – or vaccine-induced before or during pregnancy. Vaccination during pregnancy against common childhood pathogens including influenza (3, 4) and rubella (5) effectively boosts protective antibody transfer to neonates until infants reach the age of vaccination.

Despite the critical importance of antibody transfer across the placenta, and given that transfer efficiency is likely multifactorial, the mechanisms controlling it remain poorly understood. Factors known to impact transplacental antibody transfer include levels of circulating maternal antibodies, proportions of each immunoglobulin G (IgG) subclass, IgG glycosylation patterns, and placental structure, including antibody Fc receptor expression (6). Placental structure is complex and critical for proper function (7). The placental syncytiotrophoblast layer serves as the immune barrier between the maternal and fetal side and controls transcytosis of antibodies across the placenta (8). Syncytiotrophoblast express Fc receptors that bind to the antibody Fc segment in circulating antibodies in maternal blood and facilitate their transfer to the fetal circulation (1). Antibody Fc-Fc receptor interactions depend on multiple factors. Each IgG subclass has distinct affinity for Fc receptors, and glycosylation patterns further modulate binding - making this interaction a critical determinant of antibody transfer across the placenta. Acute maternal infections can also impact transplacental antibody transfer efficiency, for example SARS-CoV-2 infection compromises antibody transfer (9) and placental malaria reduces antibody transfer efficiency (10). Chronic maternal HIV infection is also associated with reduced antibody transfer efficiency, even in those who are being effectively treated with antiretroviral therapy (ART) (11, 12). A recent study found no association between HIV serostatus and histologic placental inflammation among people living with HIV (PLWH) taking ART (13), suggesting that impaired transplacental antibody transfer in PLWH may be attributable to systemic immune alterations rather than localized placental inflammation. However, the specific factors that do affect transplacental antibody transfer in PLWH, and their relative influence on the transfer process is poorly understood.

Given the ongoing knowledge gap regarding the factors that determine transplacental antibody transfer in PLWH, we investigated the impact of inflammation on the efficiency of transplacental antibody transfer in a mother-baby dyad cohort in Uganda. We measured transplacental transfer efficiency of IgG antibodies by comparing maternal and umbilical cord plasma antibody levels for 10 common pathogens. We assessed the ability of these antibodies to bind to Fc receptors, measured plasma cytokine and chemokine levels as a proxy of systemic inflammation, and analyzed placental tissue for morphological evidence of inflammation. We clustered dyads in our cohort by transplacental antibody transfer efficiency and identified two distinct clusters associated with low and high efficiency. We then investigated associations between low- and high-transplacental transfer efficiency clusters with maternal characteristics, histologic placental inflammation, and maternal and umbilical cord plasma cytokine and chemokine levels. We found that transplacental antibody transfer efficiency is associated with maternal HIV serostatus but not with histologic placental inflammatory states. Importantly, we found that elevated pro-inflammatory cytokines in maternal plasma are associated with lower antibody transplacental transfer efficiency. Our findings contribute to a greater understanding of the impact of systemic inflammation during pregnancy on transplacental antibody transfer to the fetus.

## Materials and methods

### Study site and recruitment

Participants were recruited from Mbarara Regional Referral Hospital in southwestern Uganda in 2017–2018. PLWH who reported not taking ART in the last 30 days were excluded. The study was approved by Mbarara University of Science and Technology (11/03-17), the Uganda National Council of Science and Technology (HS/2255) and Partners (now Mass General Brigham) Healthcare (2017P001319).

### Blood collection

Maternal blood was collected during labor. HIV viral load (Xpert, Cepheid, Sunnyvale, USA) and CD4+ T-cell count were measured for PLWH if not available within the last 6 months. Syphilis screening was performed on whole blood using a *Treponema pallidum* particle agglutination (TPPA) rapid test (SD Bioline Syphilis 3.0; Standard Diagnostics, Suwon City, Korea). Umbilical cord and maternal blood were spun on-site to separate plasma.

### Antigen-specific antibody isotype, subclass and FcR binding

Measles, mumps, rubella, combined herpes simplex 1 and 2 (HSV-1/2), tetanus toxoid (tetanus), hepatitis A (HepA), cytomegalovirus (CMV), Epstein-Barr virus (EBV), respiratory syncytial virus (RSV), Haemophilus influenzae type b (Hib), adenovirus type 5 hexon, and purified protein derivative (PPD)–associated antigens were coupled to MagPlex microspheres via a 2-step carbodiimide reaction. Antigen-specific IgG subclass 1, 2, 3, and 4 assays were performed in duplicate, measured using Luminex assays, and read using Intellicyt iQue, as previously described (14). Antigen-specific antibody binding assays to FcRγ2a, 2b, 3a, 3b, and FcRn were performed in duplicate, measured via Luminex multiplex assay as previously described (15).

### Cytokine measurement

ProcartaPlex™ Multiplex Immunoassays (Invitrogen, EPX200-12185-901, EPX200-12173-901) were combined and used to measure 27 chemokines and inflammatory cytokines for 289 participants in both maternal and umbilical cord plasma samples according to the manufacturer’s instructions. There was insufficient reagent to analyze samples for 60 dyads, and 3 dyads were excluded due to missing umbilical cord plasma samples. Measured cytokines and chemokines included IL-1β, IL-2, IL-4, IL-5, IL-6, IL-8, IL-10, IL-12p70, IL-13, IL-17A, IL-18, MIP-1α, granulocyte–macrophage colony-stimulating factor (GM-CSF), TNF-α, P-selectin, IL1-α, soluble intracellular adhesion molecule (sICAM)-1, E-selectin, IFN-γ, MCP-1, IFN-α, MIP-1β, SDF-α, Eotaxin, RANTES, and GROα/KC.

### Placenta collection and histopathological analysis

The placenta was collected immediately after birth in a clean container. The placentas were examined in the fresh state. Samples of membrane roll, full-thickness placental parenchyma (one from the placental center and one from the periphery), umbilical cord, and grossly identified placental lesions were cut and formalin-fixed. Once fixed, samples were processed and stained with Hematoxylin and Eosin, embedded in paraffin, and sectioned to 5μm. Histopathologic analysis was then performed by an expert perinatal pathologist (DJR) blinded to maternal HIV serostatus. Membrane rolls and umbilical cords were scored for acute chorioamnionitis (maternal and fetal inflammatory response) using the Amsterdam consensus nosology for histologic inflammation (16). A composite variable for chronic placental inflammation (CPI) was defined as histologic diagnosis of one or more of villitis of unknown etiology (VUE), chronic chorioamnionitis, plasma cell deciduitis, or chronic histiocytic intervillositis (5, 9). CPI of infectious origin was excluded. Acute chorioamnionitis (acute placental inflammation) was graded according to maternal and fetal response stages reflecting the severity of infection or inflammation on the maternal and fetal sides of the placenta, respectively. Maternal and fetal stage 2 and grades 2 or 3 were considered to represent active chorioamnionitis. Maternal vascular malperfusion (abnormal blood flow between the maternal and placental circulation) was defined as 2 or more of: infarction, retroplacental hemorrhage, distal villous hypoplasia, accelerated villous maturation, decidual arteriopathy or atherosis, trimmed placental weight <10^th^ centile, umbilical cord diameter <0.8cm, and increased syncytial knotting.

### Data analysis and visualization

Scatterplots of duplicate measurements for each antigen were reviewed, and extreme outlying values were removed as likely arising from contamination. The median phosphate-buffered saline signal (PBS) was subtracted from remaining duplicate measurements, which were then averaged to yield a single value per antigen for both cord and maternal samples. Antibody transfer ratios were calculated as the ratio of cord to maternal values.

K-means clustering was performed on the 44 transfer ratio features that had fewer than five missing observations. To determine the optimal number of clusters, we examined an elbow plot of the total within-cluster sum of squares (WCSS) for solutions ranging from k=1 to k=20. The largest reduction in the WCSS occurred between k=1 and k=2. We also evaluated average silhouette width, which was highest for the two-cluster solution, indicating that k=2 provided the best balance of within-cluster cohesion and between-cluster separation among the solutions examined. Based on these findings, we proceeded with a two-cluster solution. Cluster-defining features were examined, and clusters were designated as “low transfer ratio” and “high transfer ratio” groups based on their centroids. For each antigen, median differences between the high- and low-transfer ratio groups were visualized separately for cord and maternal plasma.

Baseline participant characteristics and placental pathology findings were compared between high and low transfer ratio groups. Continuous variables (e.g., age, birth weight) were compared using the Wilcoxon rank-sum test, and categorical variables (e.g., HIV serostatus, syphilis serostatus) were compared using Pearson’s chi-squared test.

Additional exploratory and hypothesis-generating analyses were conducted. Associations between antibody transfer ratio cluster and cytokine levels were evaluated descriptively. Cytokine concentrations below the limit of detection were imputed as the lower limit of detection, while values above the upper limit of detection were assigned the upper limit of detection. Median differences in cytokine concentrations between transfer ratio groups were computed separately for cord and maternal samples.

In addition, in a random subset of participants with glycosylation data, glycosylation features were compared between low and high transfer ratio groups using Wilcoxon rank-sum exact tests. Given the exploratory nature of this sub-analysis and the multiple comparisons performed, a Bonferroni correction was applied to adjust for multiple testing (p-values multiplied by 14, corresponding to the number of glycosylation tests conducted).

All analyses were performed using R statistical software version 4.5.1 (17). Available-case analysis was conducted; participants with missing values for variables relevant to a given analysis (e.g., antibody, cytokine, or glycosylation measurements) were excluded from that specific analysis.

## Results

### Demographic characteristics of the cohort

We analyzed maternal and umbilical cord plasma from 354 participant paired maternal/umbilical cord dyads, 176 (50%) of whom were PLWH. All participants with HIV reported taking combination ART in the last 30 days. The median age was 26 [interquartile range (IQR) 22 - 30] years. The median gestational age at birth was 39 (IQR 38 - 40) weeks.

### Defining clusters of efficiency of maternal transfer of antibodies

**Figure 1:**
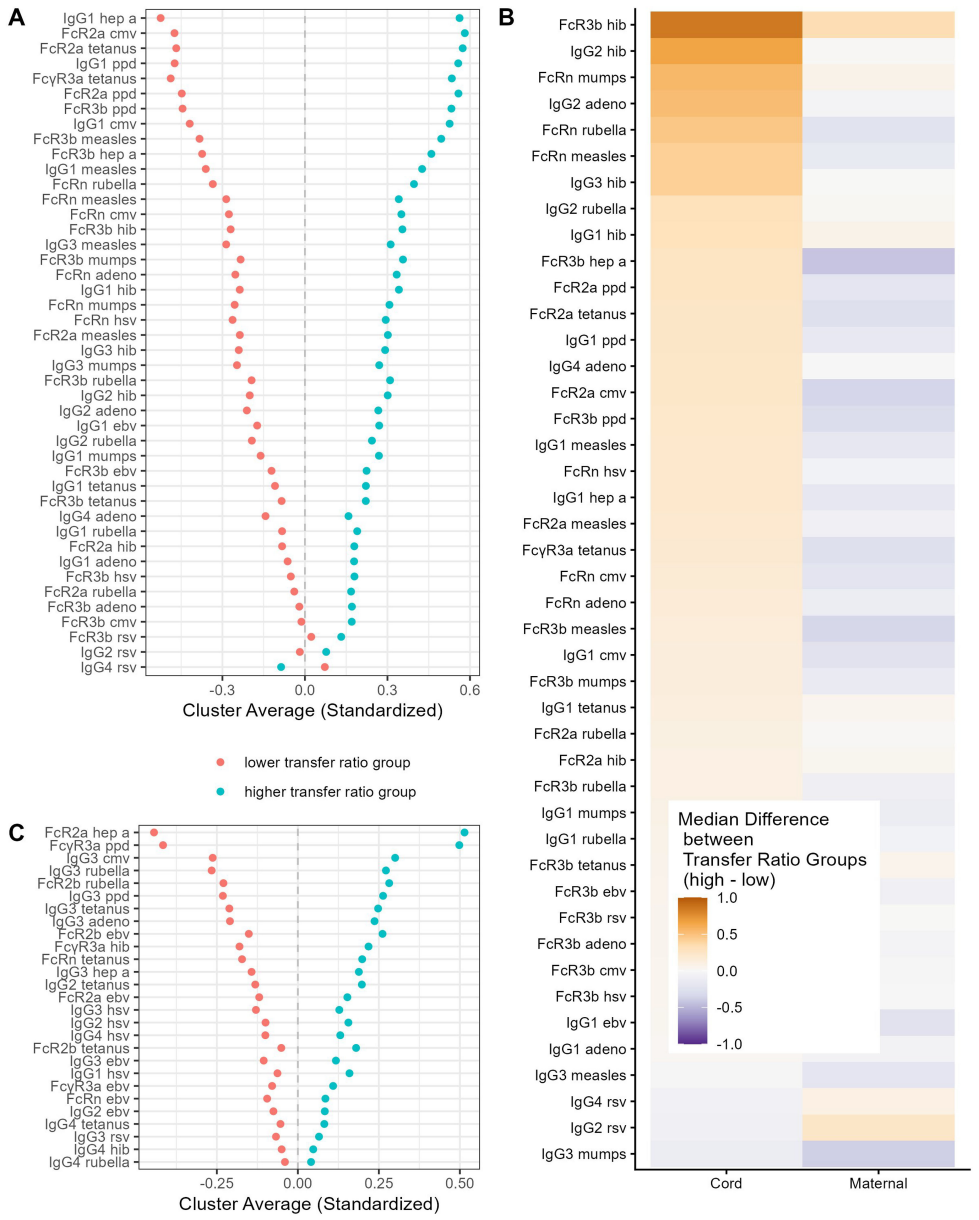
Centroids (standardized cluster average) for each antigen-specific antibody transfer ratio from a k-means clustering analysis with k=2 clusters **(A)** and Median differences in antibody levels between high and low transplacental antibody transfer ratio groups **(B)**.

In maternal and umbilical cord plasma, we measured both vaccine-induced (tetanus, rubella, mumps, measles, and hepatitis A) and naturally acquired [cytomegalovirus (CMV), *Haemophilus influenzae type B* (Hib), purified protein derivative (PPD) from tuberculosis, adenovirus, and respiratory syncytial virus (RSV)]-specific IgG antibody, FcγR and FcRn binding levels. To investigate differences in maternal transfer of antibodies across the placenta, we calculated the ratio between umbilical cord plasma and maternal plasma for all measured IgG antibody and FcR binding levels and generated a transfer ratio for each dyad in the cohort. We excluded 17 dyads with incalculable transfer ratios, where either the maternal or umbilical cord plasma values were below background-level signal. We conducted a k-means clustering analysis on the antibody transfer ratios based on 44 antibody features, after excluding features with more than 5 values missing. We identified two clusters representing high antibody transfer and low antibody transfer (**Figure 1A**). Across all 44 antigens and antibody features, transfer ratios were reduced in the lower transfer cluster, except for RSV-specific IgG4. For instance, the average IgG1 Hep a measure in the low transfer cluster was more than 0.3 SDs below the mean, whereas the average IgG1 Hep a measure in the higher transfer cluster was over 0.6 SDs above the mean. Of the 337 dyads included in the clustering analysis, 176 were in the low transfer cluster and 161 were in the high transfer cluster. As a low transfer ratio could potentially be driven by low levels in umbilical cord plasma or high levels in maternal plasma, we compared levels in both compartments between the two clusters, in order to decipher the compartment driving the transfer ratio (**Figure 1B**). We found differences in both compartments, namely in the high antibody transfer cluster, IgG and FcR binding levels were higher in umbilical cord plasma and lower in maternal plasma, while for the low transfer cluster antibody levels were lower in cord plasma and higher in maternal plasma (**Figure 1B**). This suggests that changes in both compartments contribute to the overall transfer ratio. Finally, we expanded our analysis to include all available antibody data (total 72 antibody features) to examine transfer of antibodies excluded from initial cluster analysis. This analysis revealed that the clustering of low and high transfer applied to antibody features that were not initially included in the analysis, further validating this clustering approach (**Figure 1C**).

### Association of transplacental antibody transfer ratio with maternal characteristics and placental inflammation

Next, we sought to define maternal clinical factors associated with transplacental antibody transfer efficiency. We evaluated associations between high and low antibody transfer ratios and maternal factors including age, HIV serostatus, HIV viral load, CD4+ T-cell counts and parity (**Table 1**). We found that maternal HIV serostatus and HIV viral load were associated with transplacental transfer of antibodies, as the low-transfer cluster was significantly enriched in PLWH (57% vs. 43%; p=0.01) and in those with detectable HIV viral load (>50 copies/mL, 18% vs. 7.5%, p=0.007), suggesting that active HIV infection is associated with antibody transfer across the placenta, as previously described (11) (**Table 1**). Low transplacental antibody transfer was also more common in PLWH with low CD4+ T-cell counts (p=0.06), which may reflect incomplete immune reconstitution in this population (**Table 1**). Other maternal factors including age and parity were not significantly associated with transfer efficiency (**Table 1**).

**Table 1:** Participant characteristics by antibody transfer ratio group among 337 women delivering at Mbarara Regional Referral Hospital in Uganda in 2017-2018.

Characteristic	Lower transfer ratio group n = 176^1^	Higher transfer ratio group n = 161^1^	p-value^2^
Age			0.4
Mean (SD)	26.9 (6.1)	26.1 (5.2)	
Median (Q1, Q3)	26.0 (22.5, 30.0)	26.0 (22.0, 30.0)	
HIV serostatus			0.01
Negative	76 (43%)	91 (57%)	
Positive	100 (57%)	70 (43%)	
HIV group			0.007
HIV-uninfected	76 (43%)	91 (57%)	
PPHIV, Undetectable HIV Viral Load	69 (39%)	58 (36%)	
PPHIV, Detectable HIV Viral Load	31 (18%)	12 (7.5%)	
Viral load (among HIV+), copies/mL			0.02
Mean (SD)	4,305 (14,997)	725 (4,122)	
Median (Q1, Q3)	0 (0, 221)	0 (0, 0)	
CD4+ T-cell count (among HIV+), cells/mm^3^			0.06
Mean (SD)	450 (215)	545 (315)	
Median (Q1, Q3)	425 (284, 598)	501 (338, 669)	
Parity			0.06
Primiparous	39 (22%)	45 (28%)	
Multiparous (1-4 prior deliveries)	100 (57%)	97 (60%)	
Grand multiparous (≥5 prior deliveries)	37 (21%)	19 (12%)	

^1^n (%) for categorical variables.

^2^Wilcoxon rank sum test for continuous variables and Pearson's Chi-squared test for categorical variables.

In recent literature, maternal HIV infection has not been significantly associated with placental inflammation, especially in women receiving ART (13, 18). However, the impact of acute and chronic placental inflammation on antibody transfer across the placenta is poorly defined, and there are no published studies to date on this association in PLWH. To fill this knowledge gap, we tested the association of high and low antibody transfer ratios in our cohort with acute and chronic placental inflammatory states (**Table 2**). We focused on placental inflammatory states as defined by the Amsterdam international consensus nosology (16): acute chorioamnionitis, chronic chorioamnionitis, and a composite outcome of chronic placental inflammation as defined in Methods (representative histological images are in **Supplementary Figure 1**). We did not find a significant association between any placental inflammatory states and low or high antibody transfer across the placenta in this cohort (**Table 2**). The risk of placental inflammatory states was similar between the transfer ratio groups, including villitis of unknown etiology (7% vs. 8%), acute maternal chorioamnionitis (24% vs. 25%), acute fetal chorioamnionitis (4% vs. 8%), maternal vascular malperfusion (16% vs. 16%), and fetal vascular malperfusion (9% vs. 7%). Birth weight was also similar between groups (means of 3.2 and SDs around 0.5).

**Table 2:** Placental findings by antibody transfer ratio group.

Characteristic	Lower transfer ratio group n = 176^1^	Higher transfer ratio group n = 161^1^	p-value^2^
Villitis of unknown etiology (VUE)	14 (8%)	11 (7%)	0.7
Positive screening test for syphilis	23 (13%)	13 (8%)	0.1
Acute chorioamnionitis - maternal	42 (24%)	40 (25%)	0.8
Acute chorioamnionitis - fetal	7 (4%)	13 (8%)	0.1
Maternal or fetal acute chorioamnionitis	42 (24%)	43 (27%)	0.5
Placental infarcts (≥1)	5 (3%)	7 (4%)	0.5
Maternal vascular malperfusion	28 (16%)	26 (16%)	>0.9
Fetal vascular malperfusion	15 (9%)	12 (7%)	0.7
Birth weight (kg)			0.3
Mean (SD)	3.2 (0.5)	3.2 (0.4)	
Median (Q1, Q3)	3.2 (2.9, 3.5)	3.2 (3.0, 3.5)	
Unknown	2	0	

^1^n (%) for categorical variables.

^2^Wilcoxon rank sum test for continuous variables and Pearson's Chi-squared test for categorical variables.

### Antibody glycosylation patterns are associated with efficiency of transplacental transfer

Glycosylation patterns of the Fc domain of antibodies modify their binding to Fc-receptors, thus affecting efficiency of antibody transfer across the placenta (1, 19). For example, higher galactosylated and sialylated antibodies are more efficiently transferred, while agalactosylated and bisected antibodies are transferred with low efficiency (19). To study antibody glycosylation in maternal plasma and umbilical cord plasma and association with antibody transfer efficiency, we analyzed IgG Fc glycosylation in 44 randomly selected dyads, of whom 20 were classified as low transfer ratio and 22 as high transfer ratio. We found significantly higher relative abundance of digalactosylated IgG antibodies in both maternal plasma (median 0.59 vs. 0.67) and umbilical cord plasma (median 0.62 vs. 0.68) of dyads in the high transfer ratio cluster (**Table 3**). Conversely, maternal plasma and umbilical cord plasma samples with low transfer ratio were enriched in agalactosylated IgG (median 0.17 vs 0.10 for maternal plasma; median 0.13 vs. 0.08 for umbilical cord plasma) (**Table 3**). Additionally, bisected glycan antibodies were enriched in umbilical cord plasma clustered as low antibody transfer ratio (median 0.067 vs. 0.047) (**Table 3**).

**Table 3:** Glycosylation patterns by transplacental antibody transfer ratio group among 44 women with glycosylation data.

Glycosylation	Lower transfer ratio group n = 20^1^	Higher transfer ratio group n = 22^1^	Bonferroni-corrected p-value^2^
Maternal plasma			
G0, agalactosylated	0.17 (0.10, 0.23)	0.1 (0.08, 0.12)	0.01
G1, mono galactosylated	0.22 (0.21, 0.24)	0.21 (0.19, 0.22)	0.9
G2, digalactosylated	0.59 (0.52, 0.66)	0.67 (0.64, 0.70)	0.05
G, both G1 and G2	0.82 (0.76, 0.88)	0.88 (0.86, 0.90)	0.03
S, total sialylated	0.46 (0.41, 0.50)	0.51 (0.49, 0.54)	0.08
F, fucosylated	0.48 (0.44, 0.51)	0.5 (0.48, 0.55)	>0.9
B, bisecting GlcNAc	0.075 (0.065, 0.084)	0.066 (0.060, 0.076)	0.4
Umbilical cord plasma			
G0, agalactosylated	0.13 (0.09, 0.20)	0.08 (0.08, 0.11)	0.007
G1, mono galactosylated	0.26 (0.23, 0.29)	0.22 (0.20, 0.25)	0.06
G2, digalactosylated	0.62 (0.50, 0.67)	0.68 (0.62, 0.72)	0.04
G, both G1 and G2	0.86 (0.80, 0.90)	0.91 (0.89, 0.92)	0.008
S, total sialylated	0.42 (0.34, 0.46)	0.46 (0.44, 0.50)	0.07
F, fucosylated	0.56 (0.50, 0.60)	0.6 (0.55, 0.65)	0.9
B, bisecting GlcNAc	0.067 (0.057, 0.073)	0.047 (0.031, 0.057)	0.03

^1^Median (Q1, Q3).

^2^Wilcoxon rank sum exact test, with Bonferroni correction for multiple testing.

### Association of transplacental antibody transfer ratio with maternal plasma cytokine profiles

**Figure 2:**
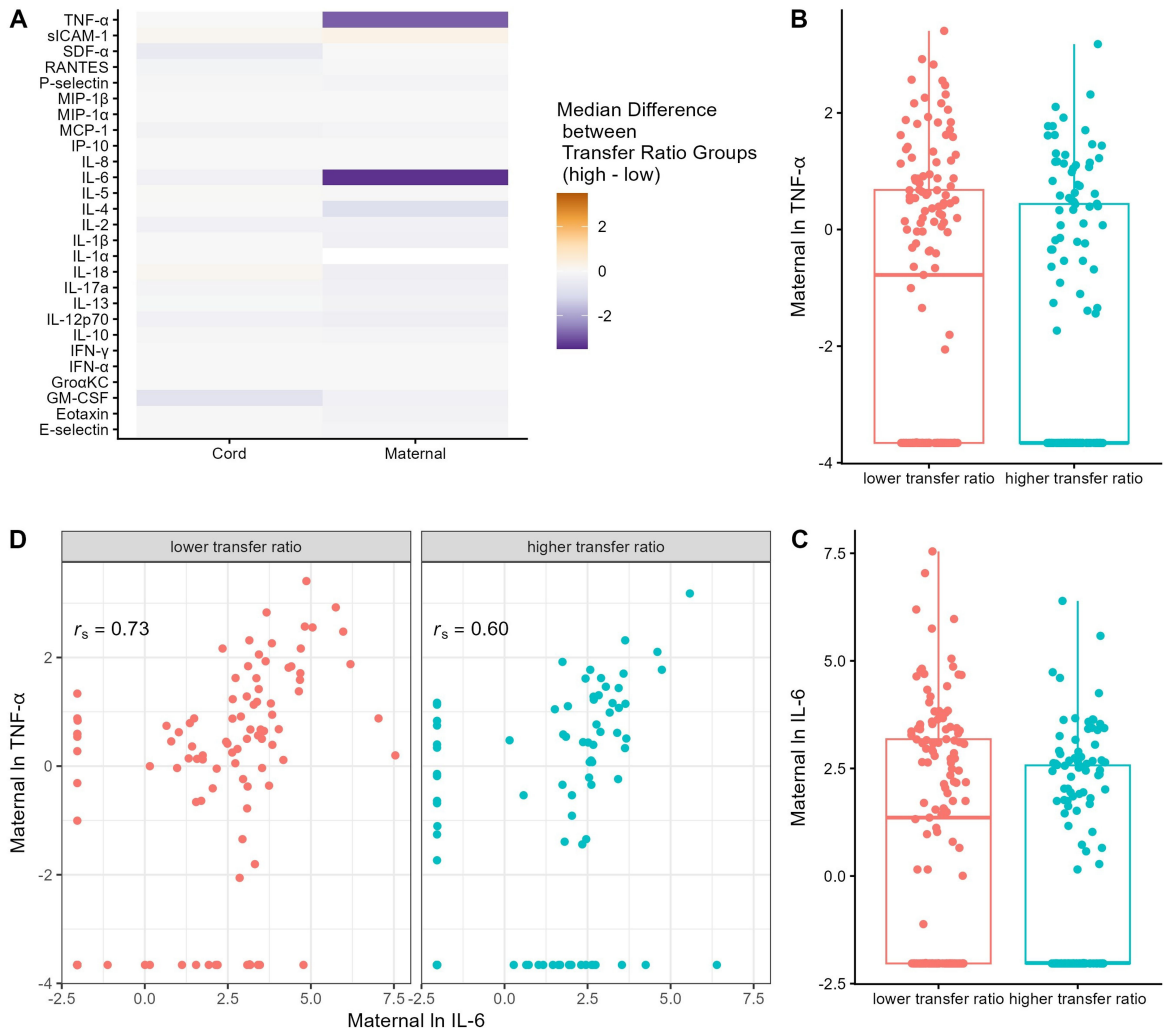
**(A)** Association between transplacental antibody transfer ratio cluster and cytokine levels among 276 women with antibody and cytokine data. **(B, C)** Association between transplacental antibody transfer ratio cluster and maternal TNF-α **(B)** and IL-6 **(C)** plasma levels among 276 women with antibody and cytokine data. **(D)** Association between maternal TNF-α and IL-6 plasma levels by transplacental antibody transfer ratio cluster among 276 women with antibody and cytokine data.

Placental inflammation is associated with elevated levels of pro-inflammatory cytokines in maternal (20–22) and umbilical cord plasma (21, 23, 24). However, it is unclear whether there is an association between the presence of pro-inflammatory cytokines and transplacental antibody transfer efficiency. In our prior study, we found minimal correlations between cytokine levels in maternal and umbilical cord plasma, suggesting that cytokine levels in these two compartments are separately regulated (12). To determine associations between placental inflammation and circulating cytokines, we analyzed cytokine and chemokine levels in maternal plasma and umbilical cord plasma of 289 women in our cohort using a multiplexed sample-sparing approach. For 276 women who had both cytokine and chemokine data and antibody data, we compared cytokine and chemokine levels in maternal plasma and umbilical cord plasma between dyads in high and low transplacental antibody transfer clusters and found no difference between clusters (**Figures 2A–C**). However, both TNF-α and IL-6 were elevated in the maternal plasma of dyads in the low transplacental antibody transfer ratios cluster (median -0.78 vs -3.66 for TNF-α, and median 1.35 vs. -2.022 for IL-6). Furthermore, levels of TNF-α and IL-6 in maternal plasma were positively correlated with each other (Spearman r = 0.73 in low-transfer group and 0.60 in high transfer group), suggesting that they may be co-regulated or produced by the same inflammatory cell population (**Figure 2D**). Overall, while antibody transfer ratios were not directly associated with histologic placental inflammation, they were impacted by circulating maternal inflammatory cytokines.

## Discussion

In this study, we analyzed a unique and large cohort of paired maternal and umbilical cord plasma samples collected in Uganda. Half of the pregnant individuals in this cohort were PLWH taking ART, reflecting current universal HIV treatment practices. We used a multiplexed sample-sparing approach to measure both vaccine-induced and naturally-acquired antibodies and their ability to bind to an array of Fc-receptors in both maternal and umbilical cord compartments. Additionally, we measured bulk antibody glycosylation in both plasma and umbilical cord plasma. Finally, we measured circulating cytokine levels in both compartments and characterized placental inflammatory states using histology. Using these comprehensive data, we sought to determine the impact of placental inflammation and systemic maternal inflammation on transplacental antibody transfer.

Transfer of antibodies across the placenta is a highly active and regulated process. While certain regulatory mechanisms have been identified, many levels of regulation remain unknown. Transfer across the placenta is performed by FcRn, the neonatal Fc receptor that binds antibodies on the maternal side of the syncytiotrophoblast layer of the placenta and transcytoses antibodies to the fetal side (25). Other IgG-binding Fc receptors are expressed on syncytiotrophoblast and other placental cells and play a role in antibody transfer (19, 26–29). Consequently, the antibody Fc fraction determines its ability to efficiently cross the placental barrier. IgG is the only antibody isotype actively transferred across the placenta (19). Moreover, IgG transfer efficiency varies across IgG subclasses. In humans, IgG1 and IgG4 transfer more efficiently than IgG3, while IgG2 transfer is minimal (30–32). Additionally, post-translational modifications of the Fc, including glycosylation, determine which antibodies are transferred to the fetus (19, 33). Transfer of antibodies across the placenta is also impacted by gestational age, total maternal IgG levels and timing of sampling relative to maternal vaccination or exposure to a certain pathogen. Thus, transplacental transfer of antibodies is a complex and multifactorial process.

Taking all these factors into account, we calculated an antibody transfer ratio for each mother-baby dyad across all measured antibody features. By analyzing antibodies specific to a wide range of viral and bacterial pathogens, representing both vaccine-induced and naturally acquired antibodies, we avoided possible skewing of the analysis due to pathogen-specific or immunization-route specific effects. We used K-means clustering to calculate 2 clusters representing low and high transfer efficiency. Using these clusters, we investigated associations between antibody transfer efficiency and maternal characteristics. Confirming our prior findings (11), we found that transplacental antibody transfer efficiency is impacted by maternal HIV serostatus and HIV viral load, as people living with HIV individuals were enriched in low-transfer efficiency cluster. However, people without HIV were also represented in the low-transfer efficiency cluster, suggesting additional underlying mechanisms. Thus, we sought to explore alternative factors impacting transplacental antibody transfer including antibody glycosylation, placental inflammation and maternal systemic inflammatory state. The associations we found between antibody glycosylation and antibody transfer efficiency recapitulated previous findings in the field (19, 33), with higher galactosylated antibodies enriched in the high transfer ratio cluster and bisected glycans enriched in the low transfer efficiency cluster.

Despite prior studies demonstrating changes in transplacental antibody transfer with acute infectious and inflammatory states, we did not find an association between acute or chronic histologic placental inflammation and transplacental antibody transfer. Further, we found that while cytokine levels in umbilical cord plasma were not associated with transplacental antibody transfer efficiency, elevated levels of the pro-inflammatory cytokines TNF-α and IL-6 in maternal plasma were associated with reduced antibody transfer efficiency. TNF-α and IL-6 are often co-expressed in response to inflammatory stimuli (34), and we found a positive correlation between circulating TNF-α and IL-6 levels in plasma samples in our cohort. Importantly, we previously showed that cytokines are highly compartmentalized, and their levels in maternal plasma and umbilical cord plasma are not correlated (12). Taken together, these findings suggest that systemic inflammation in the pregnant individual, rather than local placental inflammation, impacts transfer of antibodies to the fetus. Of note, cytokine levels in maternal serum dynamically change during labor, particularly IL-6 is induced with labor progression and returns to baseline at 4 hours postpartum, while TNF-α remains lower and more stable throughout labor (35, 36). The mechanism by which maternal inflammatory states control antibody transfer requires further mechanistic investigation. For example, circulating pro-inflammatory cytokines may impact immune cells within the placenta like placental tissue-resident macrophages called Höfbauer cells (37) or maternal macrophages and monocytes present within the decidua (38). In turn, these cells could potentially impact antibody transfer by altering their Fc receptor expression patterns and acting as a “sink” for maternal antibodies (39). The impact of maternal systemic inflammation on these cells and other potential mechanisms regulating transplacental antibody transfer requires further studies.

Our study has several limitations. Plasma cytokine levels were below or above the limit of detection for some participants, limiting our ability to analyze subtle, but meaningful, responses. Additionally, maternal blood samples were drawn during labor, which is associated with distinct cytokine and antibody levels. As a result, our analysis is not generalizable to other times in pregnancy. Furthermore, the current definitions of histologic placental inflammation are based on well-accepted histologic morphology but may not include all placental inflammatory states, including those which may occur in unique populations or on a cellular level. As a result, we may have missed important inflammatory states in our cohort. Moreover, the prevalence of histologic chronic placental inflammation in our cohort was low, and the small sample size of the population with chronic inflammatory placental lesions limited our ability to draw meaningful conclusions about associations between chronic placental inflammation, cytokine levels, and transplacental antibody transfer. In the future, larger cohorts from both low and high resource settings should be studied to explore further associations between placental inflammation and maternal antibody transfer.

Our findings demonstrate the importance of understanding regulatory mechanisms controlling transplacental antibody transfer. We found that circulating pro-inflammatory cytokines, but not histological placental inflammation, are associated with antibody transfer efficiency regardless of HIV serostatus. Understanding the factors affecting transplacental antibody transfer is of great importance, as transferred antibodies are critical in protecting newborns against infection. Future studies should relate these findings to child health outcomes and morbidity, particularly responses to vaccination and protection against infection.

## Data Availability

Data are available from the authors upon reasonable request after executing a data sharing agreement. Requests to access the datasets should be directed to lbebell@mgh.harvard.edu.
